# The Perceptions of and Factors Associated With the Adoption of the Electronic Health Record Sharing System Among Patients and Physicians: Cross-Sectional Survey

**DOI:** 10.2196/17452

**Published:** 2020-05-21

**Authors:** Martin CS Wong, Junjie Huang, Paul SF Chan, Veeleah Lok, Colette Leung, Jingxuan Wang, Clement SK Cheung, Wing Nam Wong, Ngai Tseung Cheung, Chung Ping Ho, Eng Kiong Yeoh

**Affiliations:** 1 JC School of Public Health and Primary Care Faculty of Medicine The Chinese University of Hong Kong Hong Kong; 2 Information Technology and Health Informatics Division Hospital Authority Hong Kong; 3 Information Technology Committee Hong Kong Medical Association Hong Kong

**Keywords:** electronic health records, hospital shared services, data management

## Abstract

**Background:**

The electronic health record sharing system (eHRSS) was implemented as a new health care delivery platform to facilitate two-way communication between the public and private sectors in Hong Kong.

**Objective:**

This study aimed to investigate the perceptions of and factors associated with the adoption of eHRSS among patients, the general public, and private physicians.

**Methods:**

Telephone interviews were conducted in 2018 by using a simple random sampling strategy from a list of patients who had enrolled in the eHRSS and a territory-wide telephone directory for nonenrolled residents. We completed 2000 surveys (1000 each for enrolled and nonenrolled individuals). Private physicians completed self-administered questionnaires, including 762 valid questionnaires from 454 enrolled physicians and 308 nonenrolled physicians.

**Results:**

Most participants (707/1000, 70.70%) were satisfied with the overall performance of the eHRSS. Regarding registration status, most nonenrolled patients (647/1000, 64.70%) reported that “no recommendation from their physicians and family members” was the major barrier, whereas more than half of the physicians (536/1000, 53.60%) expressed concerns on “additional workload due to use of eHRSS.” A multivariate regression analysis showed that patients were more likely to register when they reported “other service providers could view the medical records” (adjusted odds ratio [aOR] 6.09, 95% CI 4.87-7.63; *P*<.001) and “friends’ or family’s recommendation or assistance in registration” (aOR 3.51, 95% CI 2.04-6.03; *P*=.001). Physicians were more likely to register when they believed that the eHRSS could improve the quality of health care service (aOR 4.70, 95% CI 1.77-12.51; *P*=.002) and were aware that the eHRSS could reduce duplicated tests and treatments (aOR 4.16, 95% CI 1.73-9.97; *P*=.001).

**Conclusions:**

Increasing the possibility of viewing patients' personal medical record, expanding the sharable data scope for patients, and highlighting the benefits of the system for physicians could be effective to enhance the adoption of the eHRSS.

## Introduction

### Background

Health information technologies, such as electronic health record systems (eHRs), are considered to be critical in transforming health care delivery in terms of improving quality and efficiency [1,2]. In the past decade, eHRs have been launched and implemented in Western countries [3,4]. It was recognized that more extensive adoption of eHRs is effective in reducing medical errors and health care costs, enhancing medical efficacy, and improving health care delivery [5,6]. Nevertheless, the factors associated with the adoption of eHRs remained unknown, especially in Asian regions [7,8].

In Hong Kong, the Public Private Interface-electronic Patient Record (PPI-ePR) program was introduced by the hospital authority (HA) in 2006 as a new electronic platform to enhance data exchange between the public and private sectors [9]. It was the first step toward the vision to develop a territory-wide electronic health record sharing system (eHRSS) that provides a backbone to develop a two-way eHRSS and facilitates better communication between public and private health care services [10]. The eHRSS is a territory-wide health record platform funded by the Food and Health Bureau. The Information and Technology and Health Informatics Department of the HA assisted the government to develop and operate the system as a technical agency. Unlike the mandatory or opt-out enrollment models in similar health record sharing systems in other countries, for example, Denmark, the United Kingdom, or Canada, participation in the eHRSS is opt-in and on a voluntary basis for both patients and health care providers (HCPs).

The eHRSS was launched in March 2016. As of March 2019, over 1,000,000 patients, 47,000 HCPs, all private hospitals (12), and over 1400 HCPs from private sectors, including various types of clinics, elderly homes, and welfare organizations, have enrolled in the eHRSS [11]. With the satisfactory enrollment rates in general, it is an appropriate time to review the current state of the system and areas of enhancement after 3 years of its implementation.

### Objectives

This study aimed to investigate the factors associated with registration and adoption of the system among patients and physicians and to examine the awareness, acceptance, perceived benefits, and possible improvements of the eHRSS within the dual health care system of Hong Kong.

## Methods

### Recruitment

Telephone-based interviews were conducted among enrolled patients and nonenrolled residents. The survey on users was based on a list of enrolled patients provided by the HA, whereas nonusers were selected from the Hong Kong Telephone Directory, which consists of approximately 99% of land-based telephone lines. A simple random sampling methodology was adopted, and computer-generated numbers were used for subject recruitment. We assumed 65.0% as the proportion in all the outcomes. A sample size of approximately 972 enrolled participants will achieve a precision level of 0.03 from the following formula: 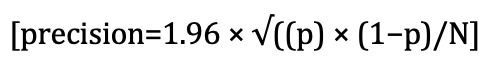, where p=proportion of outcomes. Therefore, we aimed to achieve at least 1000 successful, complete patient surveys each for enrolled and nonenrolled individuals. Assuming a refusal rate of 30%, we made more than 1500 attempts of telephone calls to complete 2000 successful surveys. The response rate was 66.67% (1000/1500) and 60.90% (1000/1642) for enrolled and nonenrolled participants, respectively.

For physician surveys, self-administered questionnaires were conducted among private physicians. Postal addresses of public institutions, nongovernment organizations, or universities were excluded. A list of all enrolled physicians in Hong Kong was provided by the electronic health record office. The response rate of physicians in previous surveys was as low as 5% [12]. To enhance the response rate, one continuous medical education (CME) point was awarded through the Hong Kong Medical Council to each completed physician response. A total of 4340 invitations were sent to private physicians through various channels, including postage, fax lines, email addresses, phone calls, lunchtime seminar programs, and high-concentration buildings where private physicians’ practices are located. In total, 762 valid questionnaires were received, consisting of 454 enrolled and 308 nonenrolled physicians. The overall response rate was 17.56% (762/4340).

### Survey Instruments

Survey items included enablers and barriers of registration in the eHRSS; the awareness, acceptance, and perceived benefits; reasons for not using the eHRSS after enrollment; and areas for service improvement. The patient and physician surveys were designed by an academic physician with relevant experience in studies related to the eHRSS and extensive expertise in clinical and public health research. The questionnaires drafted were validated by an expert panel of epidemiologists, physicians, nursing professionals, public health practitioners, and academicians. Both surveys were pilot-tested on 20 physicians and 20 patients, respectively, for feasibility and item comprehensiveness. The surveys were available in both Chinese and English versions. All surveys were anonymous. Consent was sought verbally through telephone surveys for patients and by participants’ signature through fax or postal surveys for private physicians.

### Statistical Analysis

All surveys were checked for completeness and the presence of participant consent. Data entry and analysis were performed using SPSS version 21.0 (IBM Corporation). A random check was conducted to examine the validity, quality, and accuracy of data. A descriptive analysis was performed, and the outcome variables were expressed as proportions. Two binary logistic regression models were constructed for physicians and patients. The first was to examine the predictors of registration (vs no registration), and the second was to evaluate active use (vs inactive use) of the eHRSS after registration. The predictors included (1) sociodemographic factors, (2) perceived usefulness and perceived ease of use based on the technology acceptance model [13], and (3) cues to action based on variables pertinent to the health belief model [14]. All *P* values ≤.05 were regarded as statistically significant. Variance inflation factors were calculated before the regression analysis. In patients’ analysis, 4 variables related to “perceived usefulness” were excluded because of multicollinearity, including “Keep my medical records up-to-date,” “Not necessary to bring my medical report,” “Reduce my repeated checking and information provision,” and “Physicians can get accurate and comprehensive information.” Besides, 2 interactions were found to be significant, that is, “souvenirs as an incentive” interacted with “friends’ or family’s recommendation or assistance in registration” and “the physician’s advice or assistance in registration” interacted with “friends’ or family’s recommendation or assistance in registration.” Finally, structural equation modeling (SEM) was adopted to study predictors of patients’ registration.

## Results

### Patient Surveys

#### Sociodemographic Characteristics

There were more females than males in the enrolled group (426/1000, 42.60% vs 574/1000, 57.40%) and nonenrolled group (332/1000, 33.20% vs 668/1000, 66.80%). Among the enrollees, the majority were aged between 61 and 70 years (291/1000, 29.10%), followed by 71 years or older (282/1000, 28.20%), and between 51 and 60 years (185/1000, 18.50%). Age distributions were similar in nonenrollees, with most aged between 61 and 70 years (234/1000, 23.40%), 71 years or older (229/1000, 22.90%), and between 51 and 60 years (214/1000, 21.40%; Table 1).

#### Channels of Awareness

Approximately half of the enrolled patients learned about the system from others (487/1000, 48.70%), including hospitals, clinics, health centers, district council members, and social workers. Among them, 31.80% (318/1000) learned about the system from posters or leaflets. Most nonenrollees learned about the eHRSS from television or magazine advertisements (782/1000, 78.20%) and friends and family members (165/1000, 16.50%; Multimedia Appendix 1).

#### Reasons for No Registration

The majority of nonenrollees (strongly agree or agree: 647/1000, 64.70%) agreed that no recommendations given from their physicians was the major barrier. In addition, approximately half of them (strongly agree or agree: 517/1000, 51.70%) expressed that they only visited one medical professional, and hence, registration was not required. More than one-third of them expressed concerns about the security of personal data and privacy (strongly agree or agree: 480/1000, 48.00%).

**Table 1:** Sociodemographic characteristics of patients (N=2000).

Variables		Enrollee (n=1000), n (%)	Nonenrollee (n=1000), n (%)	Total, n (%)	*P* value^a^
**Gender**					<.001
	Male	426 (42.6)	332 (33.2)	758 (37.9)	
	Female	574 (57.4)	668 (66.8)	1242 (62.1)	
**Age (years)**					<.001
	18-30	27 (2.7)	60 (6.0)	87 (4.4)	
	31-40	70 (7.0)	107 (10.7)	177 (8.9)	
	41-50	101 (10.1)	150 (15.0)	251 (12.6)	
	51-60	185 (18.5)	214 (21.4)	399 (20.0)	
	61-70	291 (29.1)	234 (23.4)	525 (26.3)	
	≥71	282 (28.2)	229 (22.9)	511 (25.6)	
	Refused to answer	44 (4.4)	6 (1.0)	50 (2.5)	
**Education**					.001
	No schooling or preschool education	92 (9.2)	80 (8)	172 (8.6)	
	Primary education	270 (27.0)	235 (23.5)	505 (25.3)	
	Junior high school	164 (16.4)	135 (13.5)	299 (15.0)	
	High school	269 (26.9)	316 (31.6)	585 (29.3)	
	Nondegree tertiary education	38 (3.8)	53 (5.3)	91 (4.6)	
	Tertiary education	120 (12.0)	155 (15.5)	275 (13.8)	
	Others	4 (0)	6 (1.0)	10 (0.5)	
	Refused to answer	43 (4.3)	20 (2.0)	63 (3.2)	
**Occupation**					.002
	Full time or part time	292 (29.2)	350 (35.0)	642 (32.1)	
	Job-waiting	7 (1.0)	10 (1.0)	17 (0.9)	
	Retirement	443 (44.3)	362 (36.2)	805 (40.3)	
	Houseworker	206 (20.6)	238 (23.8)	444 (22.2)	
	Student	13 (1.3)	17 (1.7)	30 (1.5)	
	Others	2 (0)	22 (2.2)	24 (1.2)	
	Refused to answer	37 (3.7)	1 (0)	38 (1.9)	
**Household income**					<.001
	<2000	104 (10.4)	94 (9.4)	198 (9.9)	
	2000-3999	145 (14.5)	65 (6.5)	210 (10.5)	
	4000-5999	67 (6.7)	38 (3.8)	105 (5.3)	
	6000-7999	21 (2.1)	23 (2.3)	44 (2.2)	
	8000-9999	11 (1.1)	28 (2.8)	39 (2.0)	
	10,000-14,999	71 (7.1)	60 (6.0)	131 (7)	
	15,000-19,999	50 (5.0)	63 (6.0)	113 (5.7)	
	20,000-24,999	73 (7.3)	82 (8.2)	155 (7.8)	
	25,000-29,999	35 (3.5)	57 (5.7)	92 (4.6)	
	30,000-39,999	54 (5.4)	81 (8.1)	135 (6.8)	
	40,000-59,999	43 (4.3)	40 (4.0)	83 (4.2)	
	≥60,000	48 (4.8)	25 (2.5)	73 (3.7)	
	Refused to answer	278 (27.8)	344 (34.4)	622 (31.1)	
**Joined the** **public private interface electronic patient record program**					<.001
	Yes	20 (2.0)	9 (1.0)	29 (1.5)	
	No	952 (95.2)	985 (98.5)	1937 (96.9)	
	Refused to answer	28 (2.8)	6 (1.0)	34 (1.7)	
**Required regular follow-up consultation**					<.001
	Yes	602 (60.2)	274 (27.4)	876 (43.8)	
	No	390 (39.0)	719 (71.9)	1109 (55.5)	
	Refused to answer	8 (1.0)	7 (1.0)	15 (0.8)	

^a^Proportions were compared by using chi-square tests.

#### Reasons for Not Using the System After Registration

For the enrollees who did not use the system (498 out of 1000), the reasons they did not do so after registration were “they were not sick after participation” (strongly agree or agree: 221/498, 45.5%), “they only went to one place to see a physician” (strongly agree or agree: 240/498, 49.4%), and “they did not tell the physician that they had registered (strongly agree or agree: 115/498, 23.8%).

#### Level of Satisfaction Among the Patients

Most enrollees were satisfied with the eHRSS, with 70.70% (707/1000) of the enrollees reporting that they were satisfied or strongly satisfied. Regarding the registration process, 91.20% (912/1000) of the enrollees reported that they were satisfied or strongly satisfied with the registration procedures and registration methods.

#### Perceived Areas for Future Improvement

Most of the enrollees suggested that they should be able to access their medical records through the system (30/124, 24.2%) and more sharable information (32/124, 25.8%). Others recommended that the system should be designed in a more comprehensive and user-friendly manner (23/124, 18.6%), involve the participation of more physicians (16/124, 12.9%), and increase publicity (10/124, 8.1%; Multimedia Appendix 2).

#### Factors Associated With Registration and Usage

Regarding the status of registration (Table 2), patients were more likely to register when they (1) were in the highest household income group (HK $60,000 [US $7696] or above; reference: income <14,999 [US $1924]; aOR 2.28, 95% CI 1.17-4.46; *P*=.02), (2) needed regular clinic follow-up (aOR 3.49, 95% CI 2.70-4.50; *P*<.001), (3) reported “other service providers could view the medical records” (aOR 6.09, 95% CI 4.87-7.63; *P*<.001) as perceived usefulness of the eHRSS, and (4) reported “friends’ or family’s recommendation or assistance in registration” (aOR 3.51, 95% CI 2.04-6.03; *P*=.001) as one of the cues to action.

**Table 2:** Factors associated with the status of registration and usage of the system among patients.

Variables		Status of registration		Usage of the system	
		Adjusted odds ratio (aOR; 95% CI)	*P* value	aOR (95% CI)	*P* value
**Gender**					
	Male	1 (Ref^a^)	N/A^b^	1 (Ref)	N/A
	Female	0.71 (0.55-0.91)	.008	1.18 (0.87-1.59)	.29
**Age (years)**					
	18-40	1 (Ref)	N/A	1 (Ref)	N/A
	41-60	1.00 (0.66-1.51)	.99	1.20 (0.82-3.27)	.16
	≥61	1.28 (0.79-2.06)	.32	1.73 (0.93-3.19)	.08
**Education**					
	Primary or below	1 (Ref)	N/A	1 (Ref)	N/A
	Secondary	0.96 (0.72-1.29)	.79	0.98 (0.69-1.37)	.89
	Tertiary or above	0.94 (0.62-1.43)	.79	1.22 (0.73-2.02)	.45
**Occupation**					
	Working (full time or part time)	1 (Ref)	N/A	1 (Ref)	N/A
	Not working (searching for a job, retired, houseworker, or student)	0.72 (0.52-1.01)	.06	1.05 (0.70-1.56)	.81
**Household income (HK $)**					
	≤14,999 (US $1924)	1 (Ref)	N/A	1 (Ref)	N/A
	15,000-24,999 (US $3207)	0.67 (0.47-0.97)	.03	0.70 (0.45-1.08)	.11
	25,000-59,999 (US $7696)	0.61 (0.38-0.97)	.04	0.68 (0.39-1.19)	.17
	≥60,000 (US $7696)	2.28 (1.17-4.46)	.02	0.46 (0.21-0.97)	.04
	Refused to answer	0.81 (0.59-1.10)	.18	0.84 (0.57-1.22)	.36
**Joined the** **public private interface electronic patient record program**					
	No	1 (Ref)	N/A	1 (Ref)	N/A
	Yes	2.01 (0.72-5.57)	.18	1.46 (0.56-3.81)	.44
**Required regular follow-up**					
	No	1 (Ref)	N/A	1 (Ref)	N/A
	Yes	3.49 (2.70-4.50)	<.001	1.65 (1.20-2.26)	.002
**Perceived usefulness**					
	Other medical service providers can view the medical records	6.09 (4.87-7.63)	<.001	1.71 (1.31-2.23)	<.001
**Cues to action**					
	Souvenir	1.66 (0.97-2.84)	.07	4.80 (2.72-8.48)	<.001
	Friends’ or family’s recommendation or assistance in registration	3.51 (2.04-6.03)	.001	2.07 (1.43-2.98)	<.001
	Doctor’s advice or assistance in registration	1.25 (0.85-1.84)	.27	1.52 (1.14-2.02)	.004
**Interaction effects**					
	Interaction 1^c^	0.77 (0.66-0.89)	<.001	0.64 (0.54-0.77)	<.001
	Interaction 2^d^	0.72 (0.63-0.81)	<.001	0.91 (0.84-1.00)	.04

^a^Ref: reference group in the regression analysis.

^b^N/A: not applicable.

^c^Souvenirs and friends’ or family’s recommendation or assistance in registration.

^d^Doctor’s advice or assistance in registration and friends’ or family’s recommendation or assistance in registration.

Regarding the usage of the system (Table 2), enrollees were more likely to use the system when they (1) needed regular follow-up (aOR 1.65, 95% CI 1.20-2.26; *P*=.002), (2) reported that other service providers could view the medical records (aOR 1.71, 95% CI 1.31-2.23; *P*<.001), (3) reported physicians’ advice or assistance in registration (aOR 1.52, 95% CI 1.14-2.02; *P*=.004), (4) reported friends’ or family’s recommendation or assistance in registration (aOR 2.07, 95% CI 1.43-2.98; *P*<.001), and (5) were provided with souvenirs (aOR 4.80, 95% CI 2.72-8.48; *P*<.001). The effect size of the souvenir is among the largest, followed by friends’ or family’s recommendation and the needs of regular follow-up.

SEM was adopted to study the predictors of patients’ registration (Multimedia Appendix 3). In this model, associations of observed variables to the latent variables were strong. The 2 observed variables, “friends’ or family’s recommendation or assistance” and “the physician’s advice or assistance,” had factor loadings of 0.79 and 0.66, respectively, with cues to action (latent variable). The other 4 observed variables, “reduce my repeated checking and information provision,” “keep my medical records up-to-date,” “doctors can get accurate and comprehensive information,” and “other HCPs can read my medical records,” had factor loadings between 0.93 and 0.99 with perceived benefits (latent variable). Cues to actions influenced perceived benefits with a magnitude of 0.35, and perceived benefits determined the status of registration with a magnitude of 0.38.

### Physician Surveys

#### Sociodemographic Characteristics

There were more male than female participants among the enrollees (314/454, 69.2% vs 105/454, 23.1%) and nonenrollees (216/308, 70.1% vs 64/308, 20.8%). In general, the enrollees (271/454, 59.7%; aged between 41 and 60 years) were younger than the nonenrollees (127/308, 41.2%; aged 61 years or older; Table 3).

**Table 3:** Sociodemographic characteristics of physicians (N=762).

Variables		Enrollee (n=454), n (%)	Nonenrollee (n=308), n (%)	Total, n (%)	*P* value^a^
**Gender**					.51
	Male	314 (69.2)	216 (70.1)	530 (69.6)	
	Female	105 (23.1)	64 (20.8)	169 (22.2)	
	Missing	35 (7.7)	28 (9.1)	63 (8.3)	
**Age (years)**					<.001
	≤30	3 (0.7)	0 (0)	3 (0.4)	
	31-40	47 (10.4)	14 (4.5)	61 (8.0)	
	41-50	122 (26.9)	61 (19.8)	183 (24.0)	
	51-60	149 (32.8)	86 (27.9)	235 (30.8)	
	≥61	102 (22.5)	127 (41.2)	229 (30.1)	
	Missing	31 (6.8)	20 (6.5)	51 (6.7)	
**Years of practice**					<.001
	≤4	4 (0.9)	0 (0)	4 (0.5)	
	5-9	12 (2.6)	3 (1.0)	15 (2.0)	
	10-19	107 (23.6)	50 (16.2)	157 (20.6)	
	20-29	120 (26.4)	62 (20.1)	182 (23.9)	
	≥30	178 (39.2)	170 (55.2)	348 (45.7)	
	Missing	33 (7.3)	23 (7.5)	56 (7.3)	
**Type of institution**					<.001
	Solo practice	223 (49.1)	180 (58.4)	403 (52.9)	
	With partners or group practice	150 (33.0)	70 (22.7)	220 (28.9)	
	Private hospital	31 (6.8)	13 (4.2)	44 (5.8)	
	Others	19 (4.2)	14 (4.5)	33 (4.3)	
	Missing	31 (6.8)	31 (10.1)	62 (8.1)	
**Specialty**					.006
	Nil	108 (23.8)	95 (30.8)	203 (26.6)	
	Anesthesiology	1 (0.2)	2 (0.6)	3 (0.4)	
	Community medicine	3 (0.7)	2 (0.6)	5 (0.7)	
	Emergency medicine	3 (0.7)	1 (0.3)	4 (0.5)	
	Family medicine	79 (17.4)	29 (9.4)	108 (14.2)	
	Internal medicine	61 (13.4)	18 (5.8)	79 (10.4)	
	Obstetrics and gynecology	24 (5.3)	23 (7.5)	47 (6.2)	
	Ophthalmology	13 (2.9)	7 (2.3)	20 (2.6)	
	Orthopedics and traumatology	20 (4.4)	9 (2.9)	29 (3.8)	
	Otorhinolaryngology	8 (1.8)	6 (1.9)	14 (1.8)	
	Pediatrics	24 (5.3)	21 (6.8)	45 (5.9)	
	Pathology	1 (0.2)	2 (0.6)	3 (0.4)	
	Psychiatry	8 (1.8)	24 (7.8)	32 (4.2)	
	Radiology	4 (0.9)	7 (2.3)	11 (1.4)	
	Surgery	48 (10.6)	16 (5.2)	64 (8.4)	
	Others	35 (7.7)	27 (8.8)	62 (8.1)	
	Missing	35 (7.7)	25 (8.1)	60 (7.9)	
**Joined the** **public private interface electronic patient record program**					<.001
	Yes	355 (78.2)	50 (16.2)	405 (53.1)	
	No	85 (18.7)	251 (81.5)	336 (44.1)	
	Missing	14 (3.1)	7 (2.3)	21 (2.8)	

^a^Proportions were compared by using chi-square tests.

#### Channels of Awareness

Approximately 39.4% (179/454) of the enrollees were aware of the system from peers in the health care sector, followed by practice clinics (174/454, 38.3%) and government-subsidized programs (119/454, 26.2%). For the 284 nonenrolled physicians who were aware of the system, the modes of receiving the information were as follows: mainly from peers in the health care sector (136/284, 47.9%), television or magazine advertisements (92/284, 32.4%), and posters or website (87/284, 30.6%; Multimedia Appendix 4).

#### Reasons for No Registration

More than half of the participants expressed concerns about the additional workload (strongly agree or agree: 166/308, 53.6%), whereas 45.5% (140/308) perceived the enrollment procedures to be complicated.

#### Reasons for Not Using the System After Registration

In addition, 6.8% (31/454) of enrollees did not access any patients’ medical record after the registration. Among them, 42% (13/31) stated that there was no clinical indication for accessing the data, followed by technical issues such as forgetting the log-in password (6/31, 19%) and “patients not using the system” or “patients did not inform their registration status” (6/31, 19%).

#### Level of Satisfaction Among the Physicians

Most enrollees were satisfied with the system, with 50.2% (228/454) and 7.7% (35/454) of the enrollees reporting being “satisfied” and “strongly satisfied,” respectively. A similar level of satisfaction was observed for “Instructions for use” (satisfied: 200/454, 44.1%; strongly satisfied: 35/454, 7.7%) and “compatibility of Web browser” (satisfied: 196/454, 43.2%; strongly satisfied: 34/454, 7.5%).

#### Perceived Areas for Future Improvement

Simplification of the enrollment process (enrollees: 190/454, 41.9%; nonenrollees: 166/308, 53.9%), provision of technical support (enrollees: 157/454, 34.6%; nonenrollees: 161/308, 52.3%), and improvement of interface friendliness (enrollees: 197/454, 43.4%; nonenrollees: 136/308, 44.2%) were the most commonly chosen options among physicians. Notably, over half of the enrollees (268/454, 59.0%) suggested to expand the sharable data scope (Multimedia Appendix 5), and the radiology image was the most commonly chosen option (enrollees: 335/454, 73.8%; nonenrollees: 231/308, 75%; Multimedia Appendix 6).

#### Perceived Strategies to Increase the Awareness

Traditional channels such as “television or newspaper or magazine advertisement” (enrollees: 259/454, 57.1%; nonenrollees: 112/308, 57.1%), academic publications such as medical newsletters and journals (enrollees: 168/454, 37%; nonenrollees: 161/308, 52.3%), and new media including website or social media (enrollees: 194/454, 42.7%; nonenrollees: 112/308, 36.4%) were perceived as effective strategies among the physician participants (Multimedia Appendix 7).

#### Factors Associated With Registration and Usage

Physicians were more likely to register for the eHRSS when they (1) had previously joined PPI-ePR (aOR 69.20, 95% CI 31.41-152.45; *P*<.001), (2) believed that it could improve the quality of health care service (aOR 4.70, 95% CI 1.77-12.51; *P*=.002), or (3) were aware that it could reduce duplicated tests and treatments (aOR 4.16, 95% CI 1.73-9.97; *P*=.001; Table 4).

**Table 4:** Factors associated with the status of registration among physicians.

Variables			Crude odds ratio (95% CI)	*P* value	Adjusted odds ratio (95% CI)	*P* value
**Gender**						
	Male		1 (Ref^a^)	N/A^b^	1 (Ref)	N/A
	Female		1.13 (0.79-1.61)	.51	1.15 (0.54-2.42)	.72
**Age (years)**						
	≤40		1 (Ref)	N/A	1 (Ref)	N/A
	41-60		0.52 (0.28-0.97)	.04	0.41 (0.12-1.37)	.15
	≥61		0.22 (0.12-0.43)	<.001	0.25 (0.06-1.09)	.06
**Types of medical practice**						
	Solo		1 (Ref)	N/A	1 (Ref)	N/A
	With partner or group		1.73 (1.22-2.44)	.002	1.13 (0.57-2.25)	.73
	Private hospital		1.92 (0.98-3.79)	.06	2.54 (0.60-10.81)	.21
	Others		1.10 (0.53-2.25)	.80	2.18 (0.52-9.20)	.29
**Years of practice**						
	≤9		1 (Ref)	N/A	1 (Ref)	N/A
	10-29		0.38 (0.11-1.33)	.13	1.03 (0.09-11.28)	.98
	≥30		0.20 (0.06-0.69)	.01	0.69 (0.06-8.28)	.77
**Joined the** **public private interface electronic patient record program**						
	No		1 (Ref)	N/A	1 (Ref)	N/A
	Yes		20.97 (14.27-30.81)	<.001	69.20 (31.41-152.45)	<.001
**Perceived ease of use**						
	**Timely access**					
		Disagree or strongly disagree	1 (Ref)	N/A	1 (Ref)	N/A
		Neutral	1.51 (0.80-2.84)	.20	2.03 (0.64-6.46)	.23
		Agree or strongly agree	3.48 (2.00-6.05)	<.001	2.67 (0.97-7.34)	.06
		Not applicable	0.27 (0.08-0.90)	.03	0.03 (0.01-0.24)	.001
**Cues to action**						
	**As required by subsidized program**					
		Disagree or strongly disagree	1 (Ref)	N/A	1 (Ref)	N/A
		Neutral	0.53 (0.32-0.89)	.02	0.31 (0.12-0.86)	.02
		Agree or strongly agree	0.50 (0.31-0.82)	.006	0.49 (0.20-1.20)	.12
		Not applicable	1.69 (0.95-2.99)	.07	1.72 (0.56-5.25)	.34
**Perceived benefits**						
	**Quality improvement**					
		No	1 (Ref)	N/A	1 (Ref)	N/A
		Maybe	0.79 (0.42-1.50)	.48	1.36 (0.52-3.60)	.53
		Yes	5.10 (2.75-9.44)	<.001	4.70 (1.77-12.51)	.002
	**Comprehensiveness**					
		No	1 (Ref)	N/A	1 (Ref)	N/A
		Yes	2.19 (1.59-3.01)	<.001	0.70 (0.32-1.53)	.37
	**Reduction of errors**					
		No	1 (Ref)	N/A	1 (Ref)	N/A
		Yes	1.97 (1.44-2.70)	<.001	0.57 (0.25-1.30)	.18
	**Reduction of duplicates**					
		No	1 (Ref)	N/A	1 (Ref)	N/A
		Yes	3.87 (2.72-5.51)	<.001	4.16 (1.73-9.97)	.001
	**Accuracy and timely access**					
		No	1 (Ref)	N/A	1 (Ref)	N/A
		Yes	2.52 (1.85-3.44)	<.001	1.77 (0.79-3.94)	.16
	**Disease surveillance and monitoring**					
		No	1 (Ref)	N/A	1 (Ref)	N/A
		Yes	1.48 (1.06-2.08)	.02	1.17 (0.52-2.63)	.71

^a^Ref: reference group in the regression analysis.

^b^N/A: not applicable.

Regarding the usage of the system (Table 5), insignificant results were observed for all variables in the multivariate logistic regression model. Therefore, a univariate analysis was performed to study their likelihood to use the system. Variables were reported when their *P* values were ≤.20. From Table 5, we can observe that physicians are more likely to use the system when they (1) have previously joined PPI-ePR (crude odds ratio [COR] 6.58, 95% CI 3.05-14.17; *P*<.001), (2) agreed that meeting patients’ request was a reason for enrolling in the eHRSS (COR 3.21, 95% CI 1.05-9.84; *P*=.04), (3) believed that the system could improve the quality of health care service (COR 5.34, 95% CI 1.35-21.04; *P*=.02), or (4) were aware that the system could reduce duplicated tests and treatments (COR 3.11, 95% CI 1.38-7.04; *P*<.006).

**Table 5:** Factors associated with the usage of system among physicians.

Variables			Crude odds ratio (95% CI)	*P* value
**Gender**				
	Male		1 (Ref^a^)	N/A^b^
	Female		0.52 (0.24-1.14)	.10
**Joined the** **public private interface electronic patient record program**				
	No		1 (Ref)	N/A
	Yes		6.58 (3.05-14.17)	<.001
**Perceived ease of use**				
	**Timely access**			
		Disagree or strongly disagree	1 (Ref)	N/A
		Neutral	1.25 (0.29-5.44)	.77
		Agree or strongly agree	3.57 (0.95-13.45)	.06
	**Instruction of use**			
		Disagree or strongly disagree	1 (Ref)	N/A
		Neutral	0.44 (0.12-1.59)	.21
		Agree or strongly agree	2.75 (0.60-12.61)	.19
	**Compatibility of web browser**			
		Disagree or strongly disagree	1 (Ref)	N/A
		Neutral	0.65 (0.22-1.94)	.44
		Agree or strongly agree	2.88 (0.81-10.23)	.10
**Cues to action**				
	**Patients’ request**		1 (Ref)	N/A
		Disagree or strongly disagree	1.50 (0.45-4.98)	.51
		Neutral	3.21 (1.05-9.84)	.04
		Agree or strongly agree	0.31 (0.06-1.56)	.16
**Perceived benefits**				
	**Quality improvement**			
		No	1 (Ref)	N/A
		Yes	5.34 (1.35-21.04)	.02
	**Reduction of duplicates**			
		No	1 (Ref)	N/A
		Yes	3.11 (1.38-7.04)	.006
	**Disease surveillance and monitoring**			
		No	1 (Ref)	N/A
		Yes	0.58 (0.27-1.24)	.16

^a^Ref: reference group in the regression analysis.

^b^N/A: not applicable.

## Discussion

### Principal Findings

Overall, both patients and physicians were satisfied with the eHRSS. Nonenrolled patients were aware of the system mainly from traditional communication channels (television or magazine advertisements), whereas nearly half of the enrolled patients learned about it via hospitals or clinics, community centers, district council members, and social workers. Physicians learned about the eHRSS from their peers in the health care sector. The most important factor hindering system enrollment of nonenrolled patients was the absence of recommendations from their physicians. In addition, they only visited one medical physician, and hence, registration in the system was not needed. Nonenrolled physicians were concerned about the potential increase of workload after registration and perceived the enrollment procedure as complicated. Patients did not use the system after registration mostly because they had no such need or opportunity, whereas enrolled physicians did not utilize the system as they did not perceive any clinical indication for data access.

### Explanation of Findings and Comparison With Prior Work

The survey findings reported the factors that hindered enrollment among patients. The most significant factor was the absence of recommendations from their physicians. Previous literature has demonstrated that people who appear to have authority can help a person make a particular decision [15]. Other factors included concerns about personal data and privacy issues, and the uncertainty about benefits of the system. The main reasons for not registering among physicians included perceived additional workload and complicated enrollment procedures. Evidence showed perceived workload and ease of use for a system was positively associated with its adoption [16,17]. Physicians were more likely to register when they thought that the system would improve health care quality and reduce the duplication of work, which was consistent with our findings [18]. Two reasons for not using the system among the enrolled patients were that there was no registration among physicians and that they did not inform the physicians that they had registered. As for those enrolled physicians who did not access the medical records via the system after registration, technical issues such as forgetting the log-in password were among the major reasons, and this observation is consistent with previous results [17].

Some studies have been performed on eHRs in Western countries, including the United Kingdom, the United States, and Canada [19,20]. For instance, in England, a multilevel case study with 216 participants consisting of patients, clinical staff, and project managers was conducted to investigate the use of personal electronic health records in 2010 [21]. The results showed that most of the participants perceived it neither useful nor easy to use. Nevertheless, the researchers in this case study also acknowledged that these findings should be interpreted with caution given the small sample size. As for the United States, there were 54% of physicians who adopted the eHRs in 2011 [22]. Most of the physicians who adopted an eHRs reported being satisfied with their system. Approximately half of the users agreed that the system could improve patient care. Perceived management support, provider involvement, and adequate training were the main facilitators, whereas perceived lack of usefulness and provider autonomy were the major barriers in its adoption [16]. A cross-sectional study among Canadian medical practitioners, involving 102 users and 83 nonusers, found that perceived ease of use was the strongest facilitator for eHRs use, whereas usefulness and ease of use were the main factors influencing system adoption among nonusers [23]. Although Asian countries or regions such as Japan, Taiwan, and Singapore have initiated the development of eHRs, there was a lack of studies on perceptions, awareness, and factors of adoption of the system [24].

### Strengths and Limitations

This study has comprehensively evaluated the perceptions, acceptance, and factors of eHRSS adoption, which has been implemented in Hong Kong since 2016. Although the benefits, facilitators, and barriers of eHRs have been widely discussed in Western countries, including the United Kingdom [21], Canada [23], and the United States [22], in the past decade, much effort is needed in Asian cities where eHRs were generally established in the past few years [24,25]. Meanwhile, previous studies mainly focused on either patients’ or physicians’ perspectives [26,27]. Our study included perceptions among enrolled patients, nonenrolled patients, enrolled physicians, and nonenrolled physicians. In addition, an updated patient list that contained enrolled patients and a territory-wide telephone directory for nonenrolled patients were used with a simple random sampling strategy, which enhanced the generalizability of our findings.

There are several limitations of this study. First, the survey was a cross-sectional study and could not establish a cause-and-effect relationship because of the possibility of reverse causality. Prospective longitudinal studies are required to confirm the facilitators and barriers. In addition, the survey questions were designed through face validity rather than construct validity. The consistency reliability of the survey measurements was yet to be evaluated. In addition, the overall response rate among physician participants was low (17.6%), and it might have caused nonresponse bias. However, the study adopted different strategies to enhance the response rate, including CME point, postage, fax lines, email addresses, phone calls, lunchtime seminar programs, and visits to high-concentration buildings where private physicians’ practices are located. Hence, the response rate was much higher than that in the previous local study (5%). Finally, there may be other variables that could affect the registration and adoption of eHRSS, and hence, some residual confounders may remain uncontrolled.

### Lessons Learned

Findings of this study can inform future clinical practice and public health policy on the promotion of eHRSS adoption. To enhance the enrollment rate of eHRSS among patients who have not yet registered, recommendations by primary care physicians during their daily clinical practice is considered to be the most influential factor. It is also important to deliver a sense of adequate and appropriate security protection to the public because it is another key concern for the adoption of eHRSS among patients [28,29]. Multilevel measurements are needed to protect personal data in the eHRSS, such as consent-based record sharing, role-based access control, full data encryption, as well as network and application security defense and protection. In addition, the awareness of the benefits of the eHRSS should be enhanced in the community. To achieve this, future promotional campaigns and educational seminars on the benefits of eHRSS can be effective based on findings from previous evaluations [16]. As for the primary care physicians, communication among the physician users may influence the use of eHRSS. The study found that physician users learned about the system most commonly from their peers in the health care sectors. Therefore, more interviews of the enrolled physicians in electronic health (eHealth) news and booths in physicians’ conferences could be organized to promote the adoption of eHRSS among them [30]. To enhance the actual use of the eHRSS after enrollment among patients, efforts to improve the enrollment among physicians can be effective as it was found to be the most significant factor associated with its use. For physicians who have already enrolled in the eHRSS, it is suggested to provide easier channels for them to retrieve passwords in case they were forgotten. In addition, more technical support on the system could be provided and the user-friendliness of the system interface could be enhanced to maintain long-term adoption of the eHRSS by reducing the time spent on dealing with technical issues.

### Conclusions

Participants were satisfied with the overall performance of the system. For patients, the possibility of viewing their personal medical records and expanding the sharable data scope in the system could be a future direction of development. In addition, messages about the stringent measures in protecting privacy and benefits of the system should be clearly conveyed to the public. For physicians, major barriers of registration and usage, such as perceived additional workload, complicated procedures, and lack of technical assistance, will require additional practical and logistic support. It is recommended to enlist enrolled physicians to promote the system among their peer colleagues, such as more interviews in eHealth news and booths in physicians’ conferences.

## Appendix

Multimedia Appendix 1 Channels for patients to know about electronic health record sharing system.

Multimedia Appendix 2 Perceived areas to improve electronic health record sharing system among patients.

Multimedia Appendix 3 Factors associated with electronic health record sharing system registration among patients: structural equation modeling .

Multimedia Appendix 4 Channels for physicians to know about electronic health record sharing system.

Multimedia Appendix 5 Perceived areas to improve electronic health record sharing system among physicians.

Multimedia Appendix 6 Perceived scope of areas to be expanded among physicians.

Multimedia Appendix 7 Perceived strategies to increase the awareness of electronic health record sharing system among physicians.

## Abbreviations

aOR: adjusted odds ratio
CME: continuous medical education
COR: crude odds ratio
eHealth: electronic health
eHRs: electronic health record systems
eHRSS: electronic health record sharing system
HA: hospital authority
HCP: health care provider
PPI-ePR: Public Private Interface-electronic Patient Record
SEM: structural equation modeling
