# Supplementary material for: The Perceptions of and Factors Associated With the Adoption of the Electronic Health Record Sharing System Among Patients and Physicians: Cross-Sectional Survey
Source: JMIR Med Inform. 2020 May 21;8(5):e17452. doi: 10.2196/17452 (PMC7273237; doi:10.2196/17452)
Supplement: Multimedia Appendix 1 [file medinform_v8i5e17452_app1.pdf]

Multimedia Appendix 1. Channels for patients to know about electronic health record sharing system.

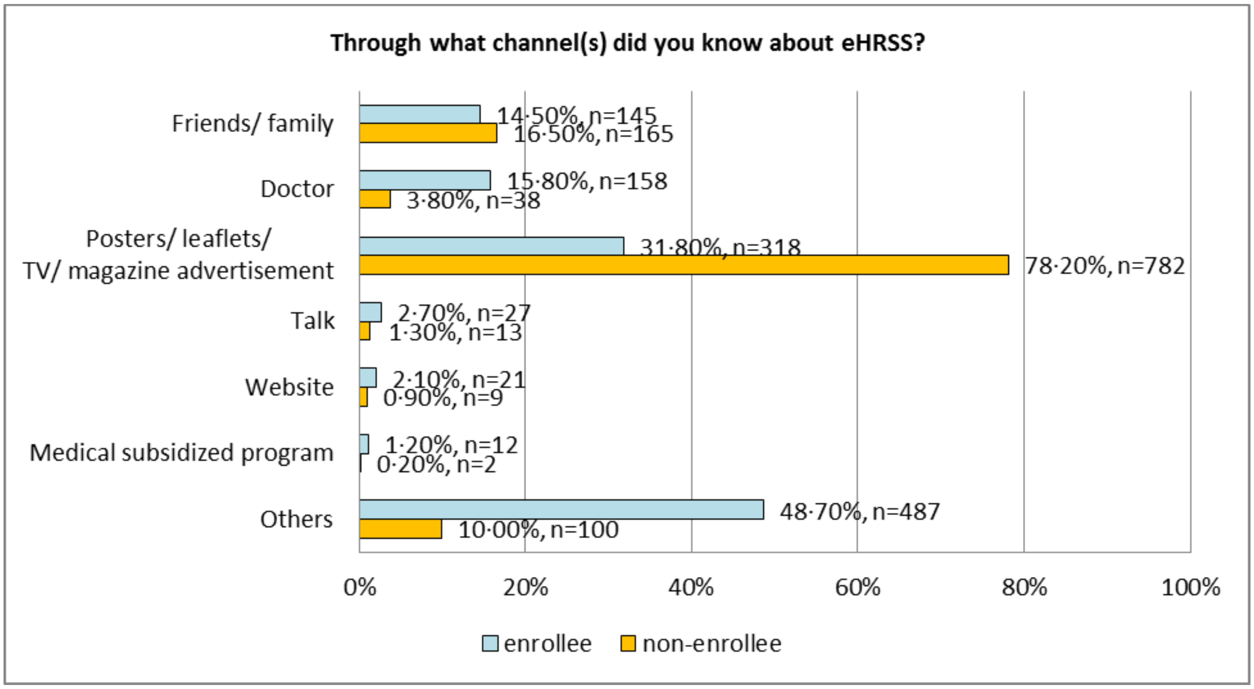

Base: Enrollee = 1,000; Non-enrollee = 1,000

Note: Multiple answers were allowed. Others included “Hospital/ Clinic”, “Health/ Community/ Elderly center”, “Social worker”, “District council member”, “Non-profit Organizations”, “Maternal & Child Health Centre”.
