# Supplementary material for: The Perceptions of and Factors Associated With the Adoption of the Electronic Health Record Sharing System Among Patients and Physicians: Cross-Sectional Survey
Source: JMIR Med Inform. 2020 May 21;8(5):e17452. doi: 10.2196/17452 (PMC7273237; doi:10.2196/17452)
Supplement: Multimedia Appendix 2 [file medinform_v8i5e17452_app2.pdf]

Multimedia Appendix 2. Perceived areas to improve electronic health record sharing system among patients.

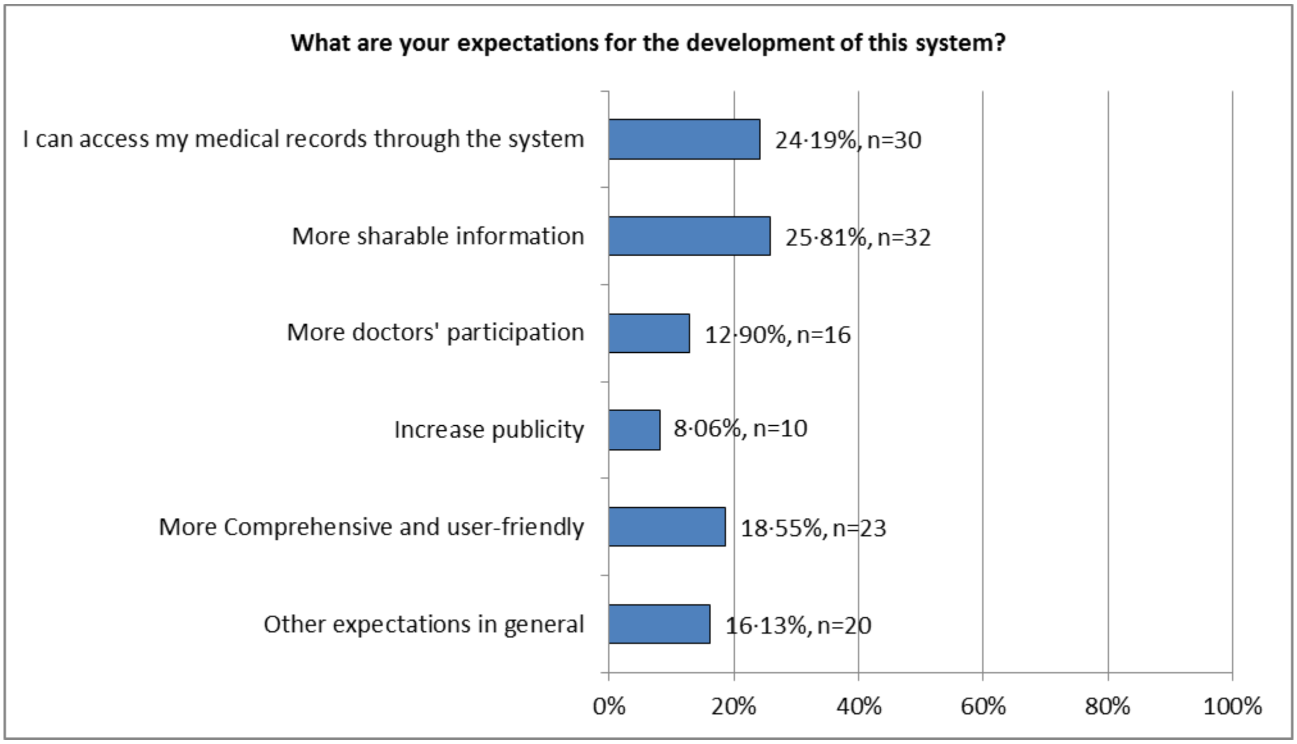

Base: Enrollee who reported to have expectations = 124

Note: Multiple answers were allowed. Other expectations in general included "Continuous improvement", "Reminder of appointment", "Available to elderly center", "Improve registration process", "Hope to update".
