# Supplementary material for: The Perceptions of and Factors Associated With the Adoption of the Electronic Health Record Sharing System Among Patients and Physicians: Cross-Sectional Survey
Source: JMIR Med Inform. 2020 May 21;8(5):e17452. doi: 10.2196/17452 (PMC7273237; doi:10.2196/17452)

Multimedia Appendix 3. Factors associated with electronic health record sharing system registration among patients: structural equation modeling .

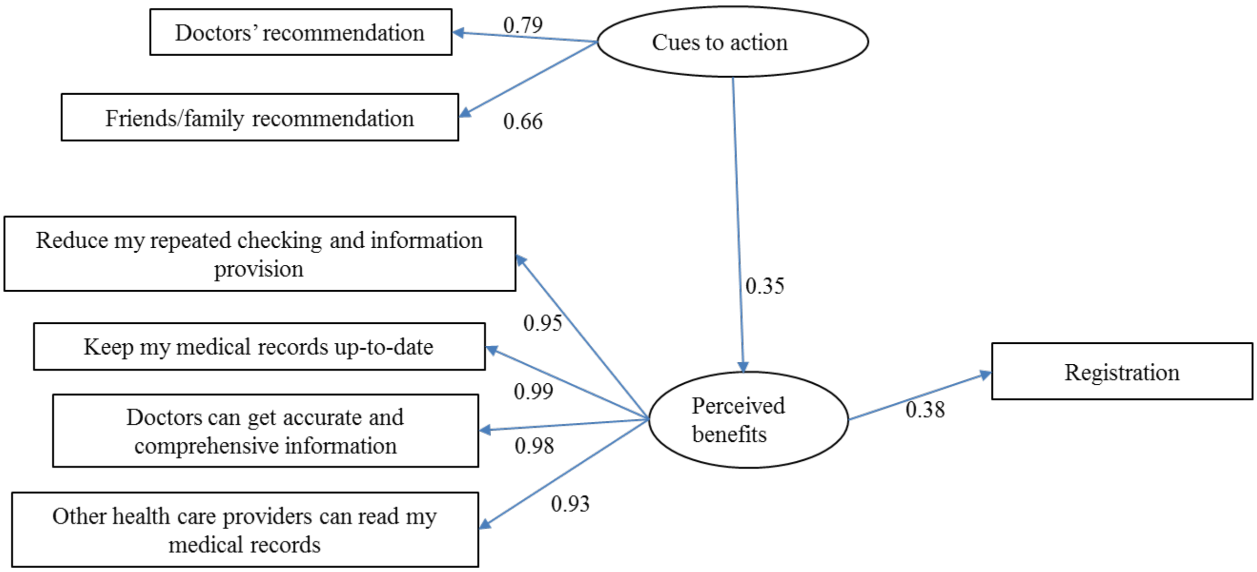

Supplement: Multimedia Appendix 3 [file medinform_v8i5e17452_app3.pdf]
