# Supplementary material for: The Perceptions of and Factors Associated With the Adoption of the Electronic Health Record Sharing System Among Patients and Physicians: Cross-Sectional Survey
Source: JMIR Med Inform. 2020 May 21;8(5):e17452. doi: 10.2196/17452 (PMC7273237; doi:10.2196/17452)
Supplement: Multimedia Appendix 4 [file medinform_v8i5e17452_app4.pdf]

Multimedia Appendices

Multimedia Appendix 1. Channels for patients to know about electronic health record sharing system.

Multimedia Appendix 2. Perceived areas to improve electronic health record sharing system among patients.

Multimedia Appendix 3. Factors associated with electronic health record sharing system registration among patients: structural equation modeling .

Multimedia Appendix 4. Channels for physicians to know about electronic health record sharing system.

Multimedia Appendix 5. Perceived areas to improve electronic health record sharing system among physicians.

Multimedia Appendix 6. Perceived scope of areas to be expanded among physicians.

Multimedia Appendix 7. Perceived strategies to increase the awareness of electronic health record sharing system among physicians.

Multimedia Appendix 1. Channels for patients to know about electronic health record sharing system.

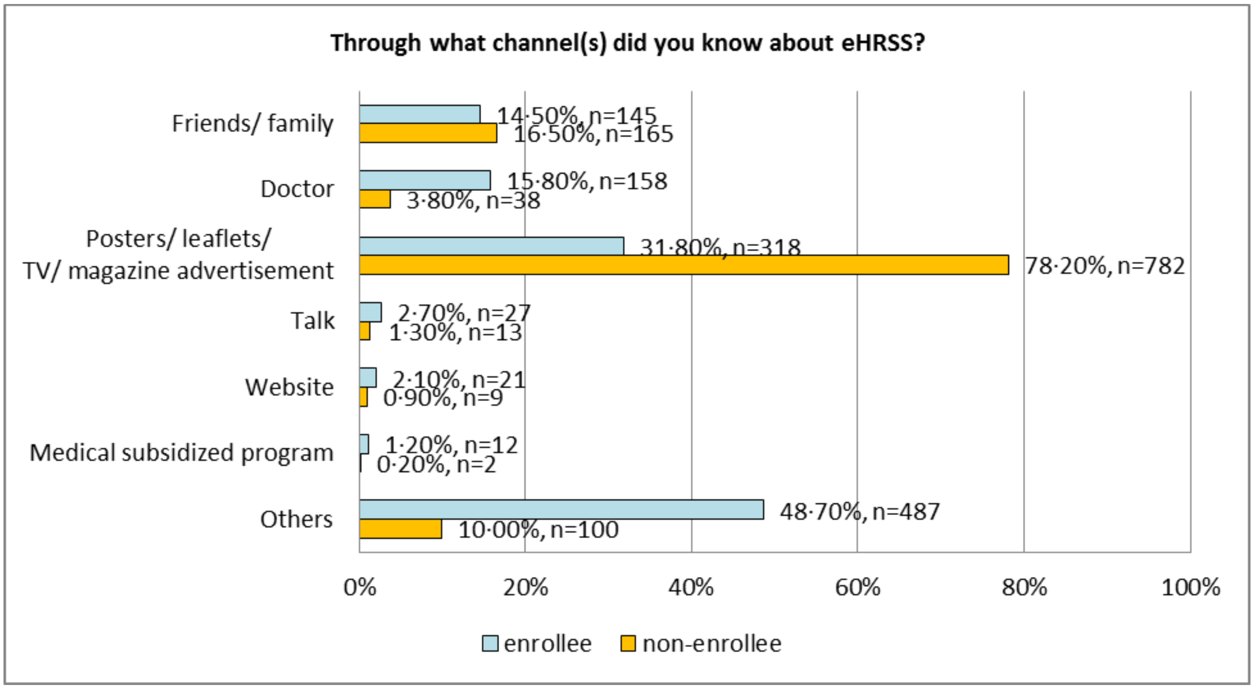

Base: Enrollee = 1,000; Non-enrollee = 1,000

Note: Multiple answers were allowed. Others included “Hospital/ Clinic”, “Health/ Community/ Elderly center”, “Social worker”, “District council member”, “Non-profit Organizations”, “Maternal & Child Health Centre”.

Multimedia Appendix 2. Perceived areas to improve electronic health record sharing system among patients.

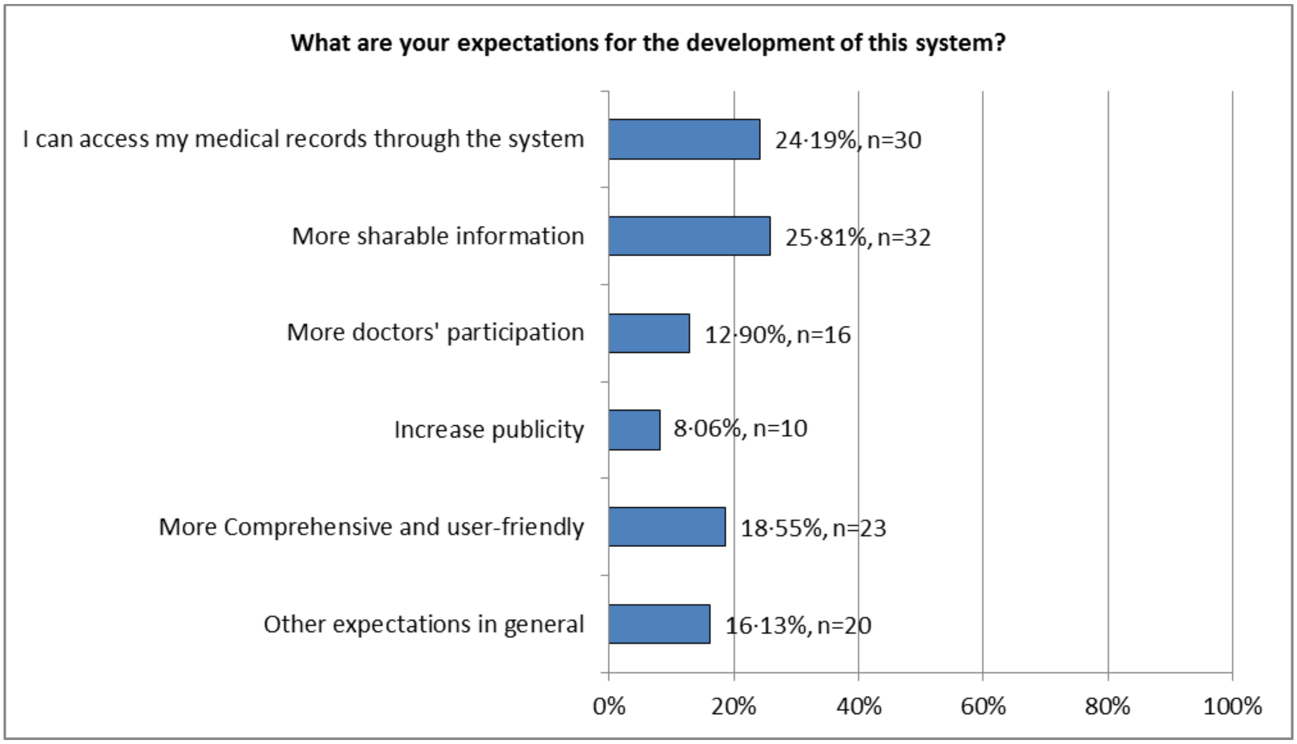

Base: Enrollee who reported to have expectations = 124

Note: Multiple answers were allowed. Other expectations in general included “Continuous improvement”, “Reminder of appointment”, “Available to elderly center”, “Improve registration process”, “Hope to update”.

Multimedia Appendix 3. Factors associated with electronic health record sharing system registration among patients: structural equation modeling .

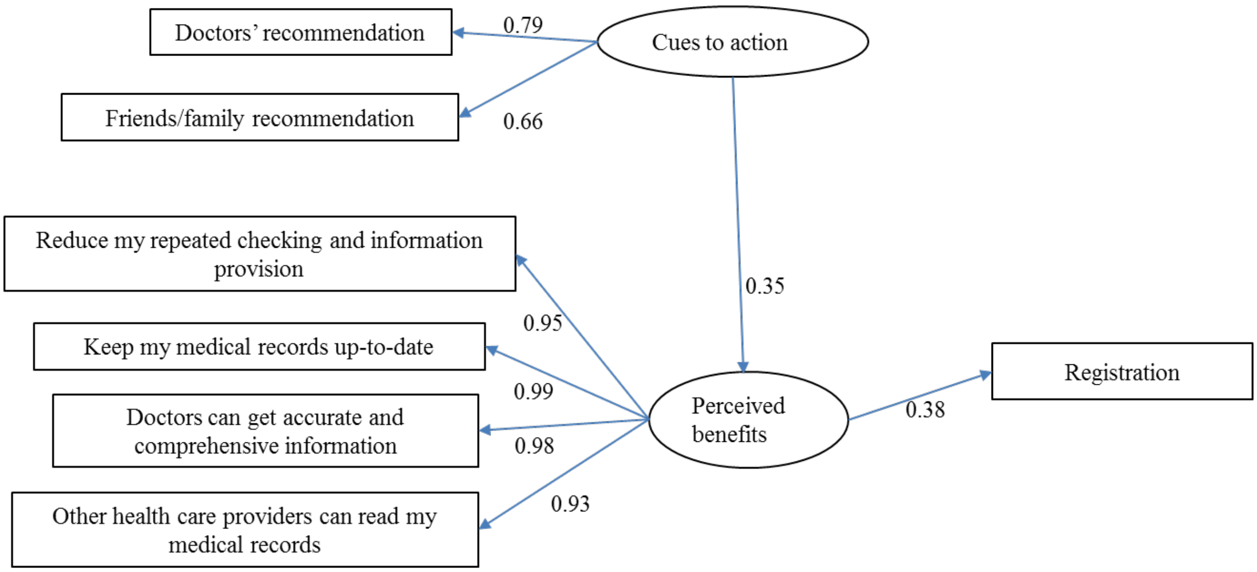

Multimedia Appendix 4. Channels for physicians to know about electronic health record sharing system.

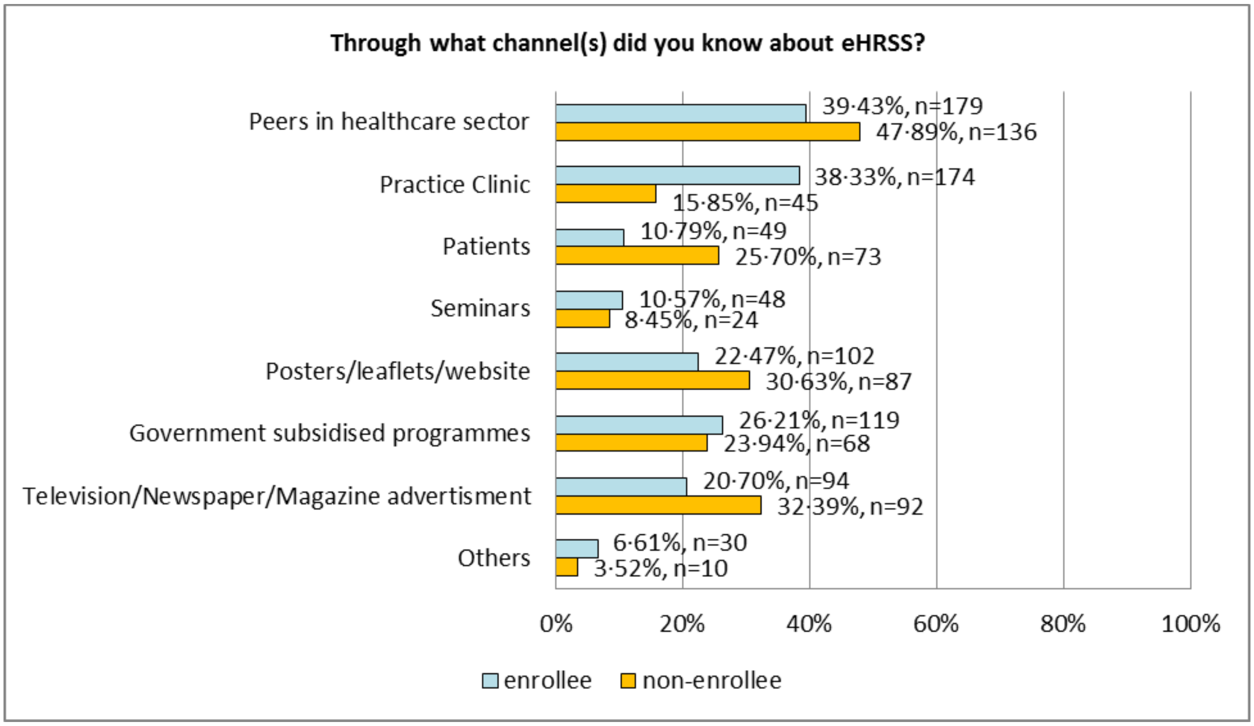

Base: Enrollee = 454; Non-enrollee = 284
Note: Multiple answers were allowed. Others included “Worked in HA before”, “PPI-ePR user”, “Government invitation letter”, “HKMA”.

Multimedia Appendix 5. Perceived areas to improve electronic health record sharing system among physicians.

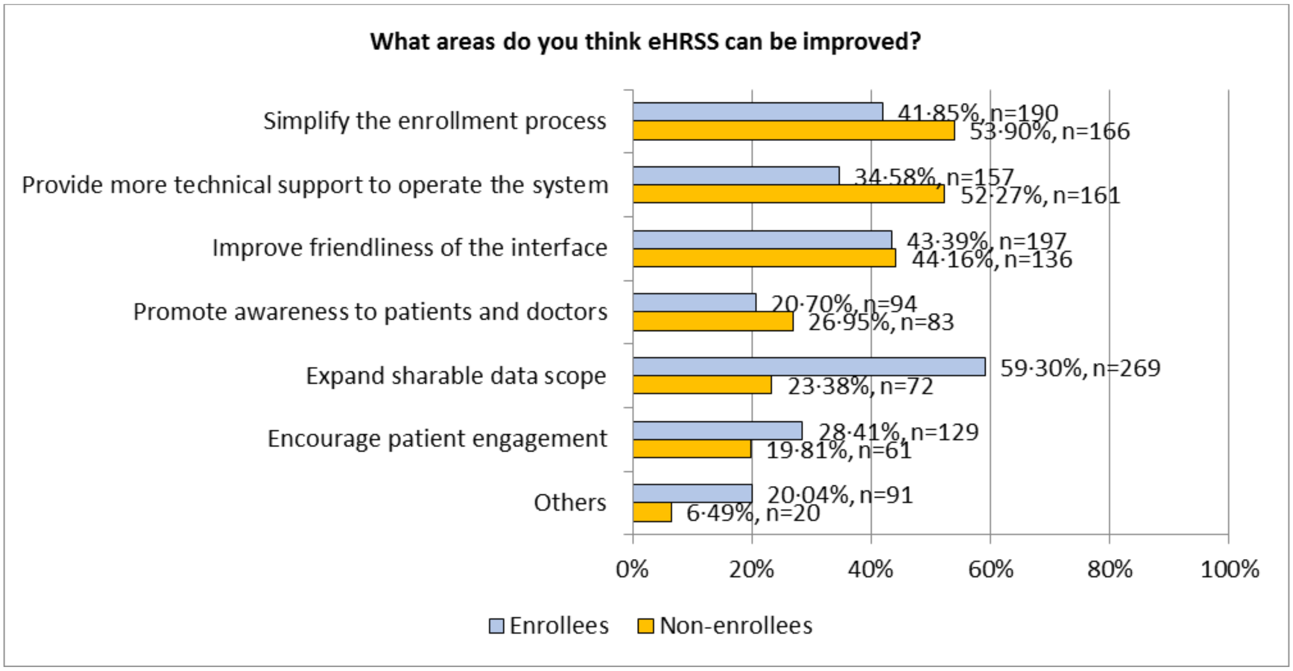

Base: Enrollee = 454; Non-enrollee = 308

Note: Multiple answers were allowed. Others included “More browser compatibility”, “Increase the speed of data upload to system”, “Extend time-out duration”, “Show images e.g. CT, x-rays, ECG, MRI films”.

Multimedia Appendix 6. Perceived scope of areas to be expanded among physicians.

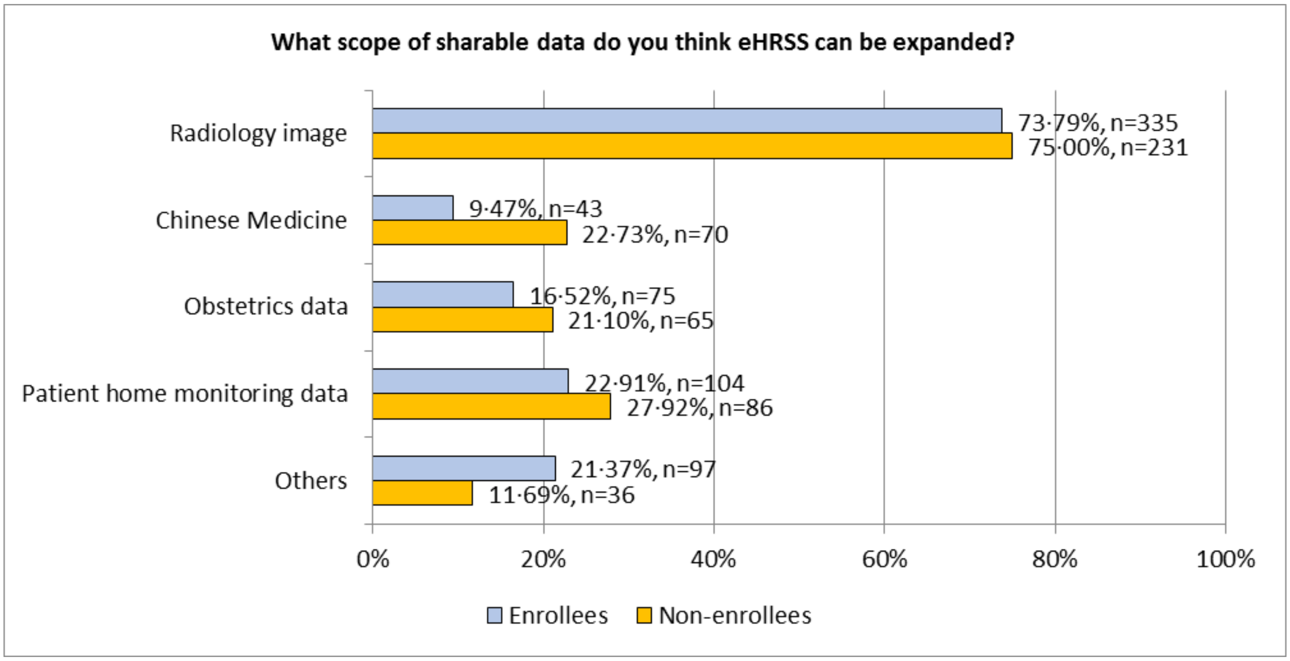

Base: Enrollee = 454; Non-enrollee = 308

Note: Multiple answers were allowed. Others included "Outpatient record", "Clinic consultation notes", "Operation record".

Multimedia Appendix 7. Perceived strategies to increase the awareness of electronic health record sharing system among physicians.

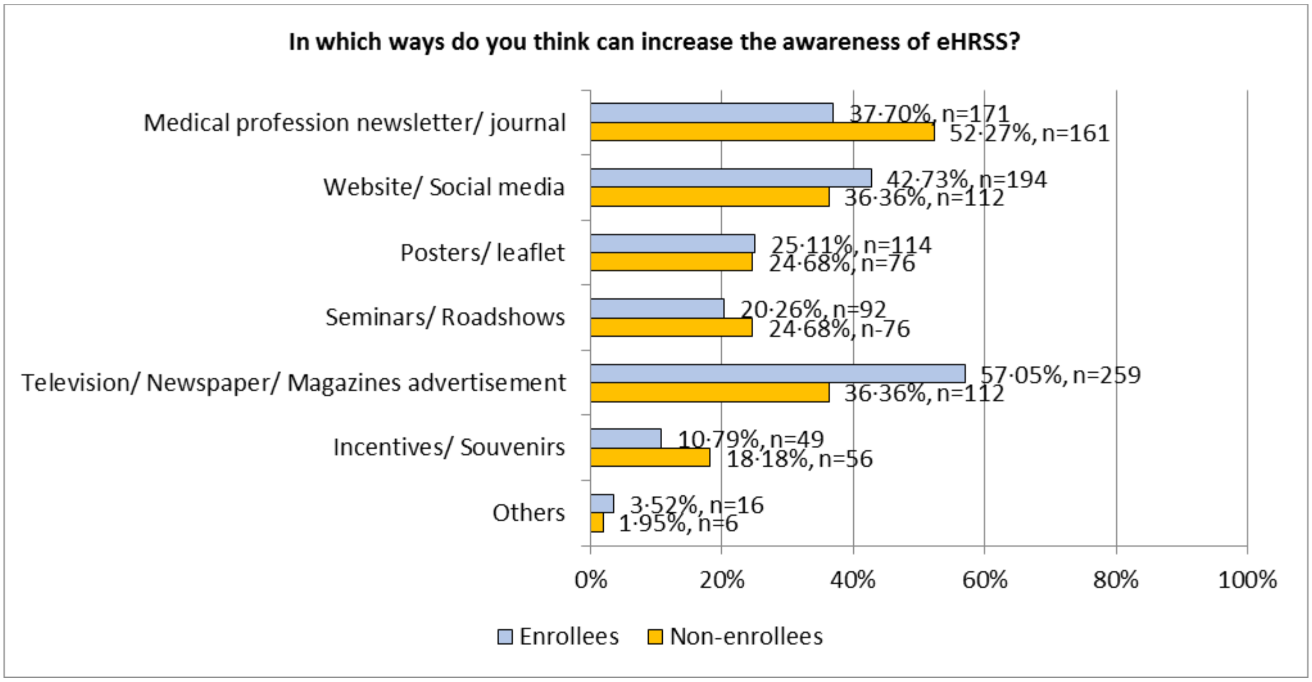

Base: Enrollee = 454; Non-enrollee = 308

Note: Multiple answers were allowed. Others included "through colleges", "more pop-up station at hospital area for instant registration", "more friendly system".
