# Supplementary material for: The Perceptions of and Factors Associated With the Adoption of the Electronic Health Record Sharing System Among Patients and Physicians: Cross-Sectional Survey
Source: JMIR Med Inform. 2020 May 21;8(5):e17452. doi: 10.2196/17452 (PMC7273237; doi:10.2196/17452)
Supplement: Multimedia Appendix 5 [file medinform_v8i5e17452_app5.pdf]

Multimedia Appendix 5. Perceived areas to improve electronic health record sharing system among physicians.

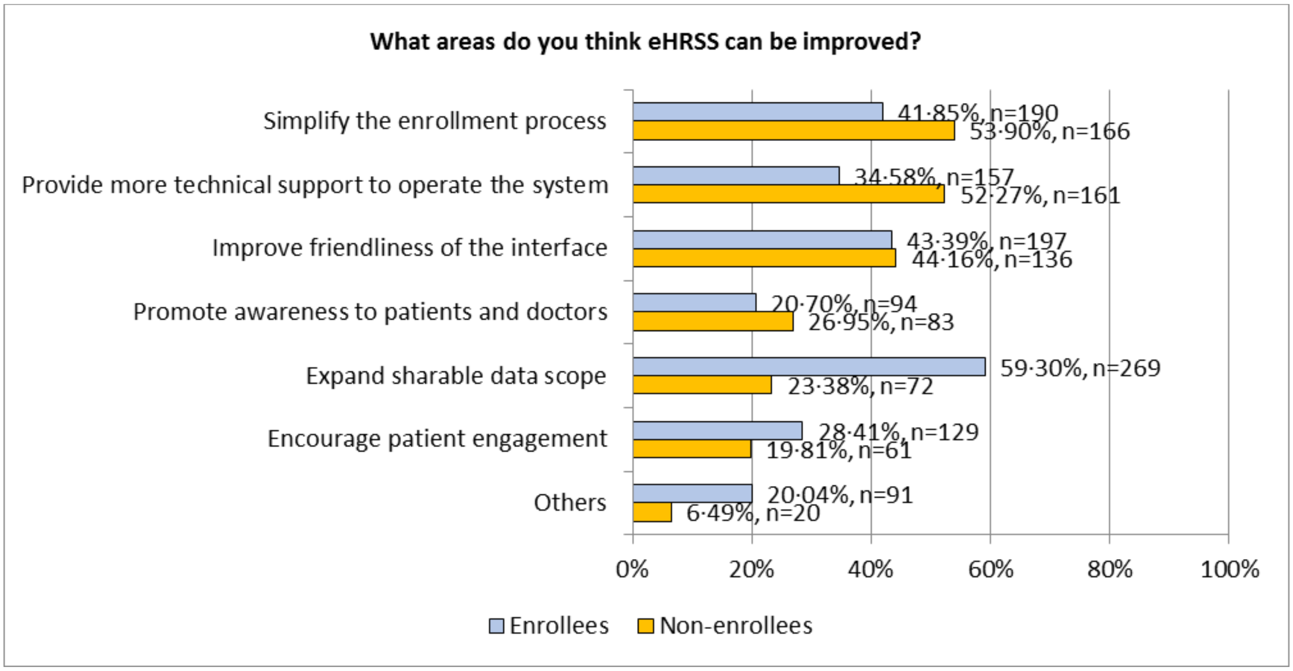

Base: Enrollee = 454; Non-enrollee = 308

Note: Multiple answers were allowed. Others included “More browser compatibility”, “Increase the speed of data upload to system”, “Extend time-out duration”, “Show images e.g. CT, x-rays, ECG, MRI films”.
