# Supplementary material for: The Perceptions of and Factors Associated With the Adoption of the Electronic Health Record Sharing System Among Patients and Physicians: Cross-Sectional Survey
Source: JMIR Med Inform. 2020 May 21;8(5):e17452. doi: 10.2196/17452 (PMC7273237; doi:10.2196/17452)
Supplement: Multimedia Appendix 7 [file medinform_v8i5e17452_app7.pdf]

Multimedia Appendix 7. Perceived strategies to increase the awareness of electronic health record sharing system among physicians.

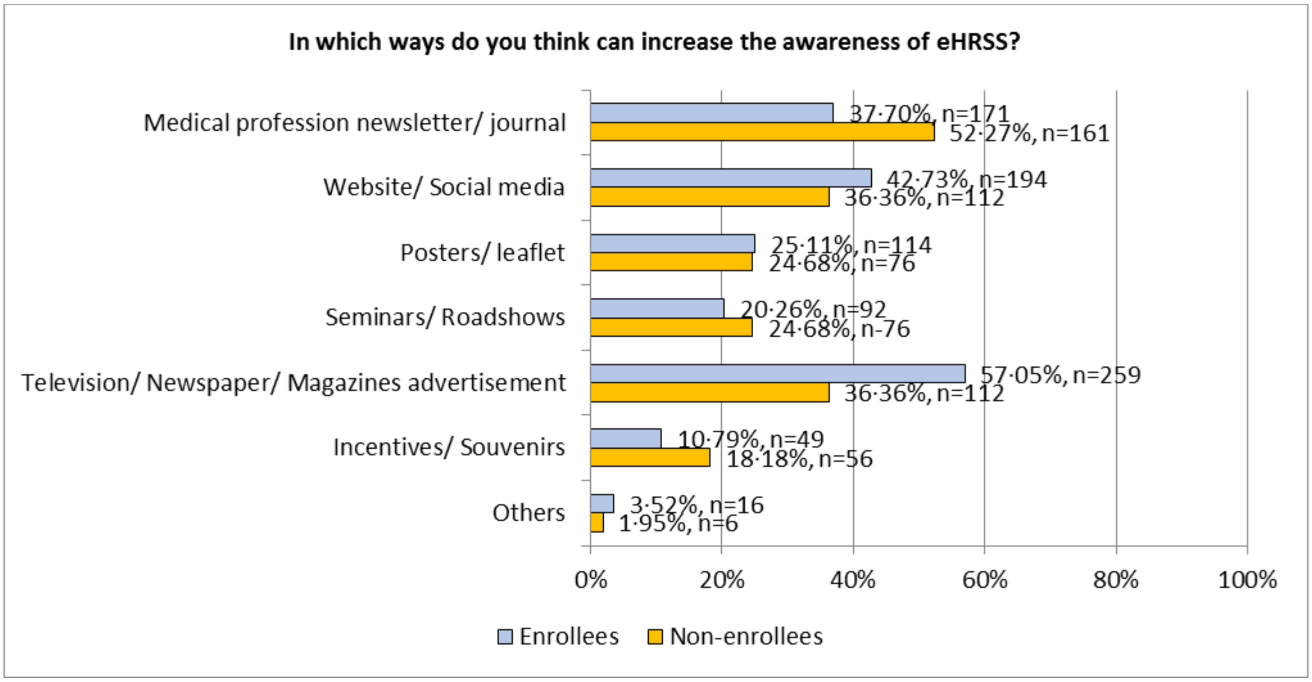

Base: Enrollee = 454; Non-enrollee = 308

Note: Multiple answers were allowed. Others included "through colleges", "more pop-up station at hospital area for instant registration", "more friendly system".
